# Supplementary material for: Tizoxanide Antiviral Activity on Dengue Virus Replication
Source: Viruses. 2023 Mar 7;15(3):696. doi: 10.3390/v15030696 (PMC10055917; doi:10.3390/v15030696)
Supplement: Supplementary file 1 [file viruses-15-00696-s001.zip › Additional file 1 S1.pdf]

Table S1: Pre-treatment test

	[ ] µM	PFU	PFU	PFU	Average	Standard deviation	% VI
1 h	**0**	285	288	284	286	1.70	
	**0.30**	287	286	285	286	0.82	**-0.1**
	**0.40**	286	284	283	284	1.25	**0.5**
	**0.50**	280	282	282	281	0.94	**1.5**
	**0.60**	283	284	282	283	0.82	**0.9**
12 h	**0**	280	285	282	282	2.05	
	**0.1**	278	275	277	277	1.25	**2.0**
	**1.4**	279	274	277	277	2.05	**2.0**
	**1.8**	282	280	278	280	1.63	**0.8**
	**2.2**	275	270	277	274	2.94	**3.0**
24 h	**0**	282	280	285	282	2.05	
	**0.30**	280	283	286	283	2.45	**-0.2**
	**0.40**	284	280	283	282	1.70	**0.0**
	**0.50**	281	277	284	281	2.87	**0.6**
	**0.60**	280	277	275	277	2.05	**1.8**
