# Supplementary material for: Tizoxanide Antiviral Activity on Dengue Virus Replication
Source: Viruses. 2023 Mar 7;15(3):696. doi: 10.3390/v15030696 (PMC10055917; doi:10.3390/v15030696)
Supplement: Supplementary file 1 [file viruses-15-00696-s001.zip › Additional file 1 S2.pdf]

Table S2: Viral adsorption inhibition assay

**[ ] μM**	**PFU**	**PFU**	**PFU**	**Average**	**Standard deviation**	**% VI**
**0.0**	253	255	258	255	2.05	
**1.0**	254	252	257	254	2.05	**0.4**
**1.4**	256	252	250	253	2.49	**1.0**
**1.8**	260	255	256	257	2.16	**-0.7**
**2.2**	255	251	254	253	1.70	**0.8**
