# Supplementary material for: Tizoxanide Antiviral Activity on Dengue Virus Replication
Source: Viruses. 2023 Mar 7;15(3):696. doi: 10.3390/v15030696 (PMC10055917; doi:10.3390/v15030696)
Supplement: Supplementary file 1 [file viruses-15-00696-s001.zip › Additional file 1 S3.pdf]

Table S3: Virus penetration inhibition assay

[ ] μM	PFU	PFU	PFU	Average	Standard deviation	% VI
**0.0**	250	240	247	246	4.19	
**1.0**	250	255	249	251	2.62	**-2.3**
**1.4**	240	247	251	246	4.55	**-0.1**
**1.8**	245	252	250	249	2.94	**-1.4**
**2.2**	240	248	245	244	3.30	**0.5**
