# Supplementary material for: Tizoxanide Antiviral Activity on Dengue Virus Replication
Source: Viruses. 2023 Mar 7;15(3):696. doi: 10.3390/v15030696 (PMC10055917; doi:10.3390/v15030696)
Supplement: Supplementary file 1 [file viruses-15-00696-s001.zip › Figure S1 new.pdf]

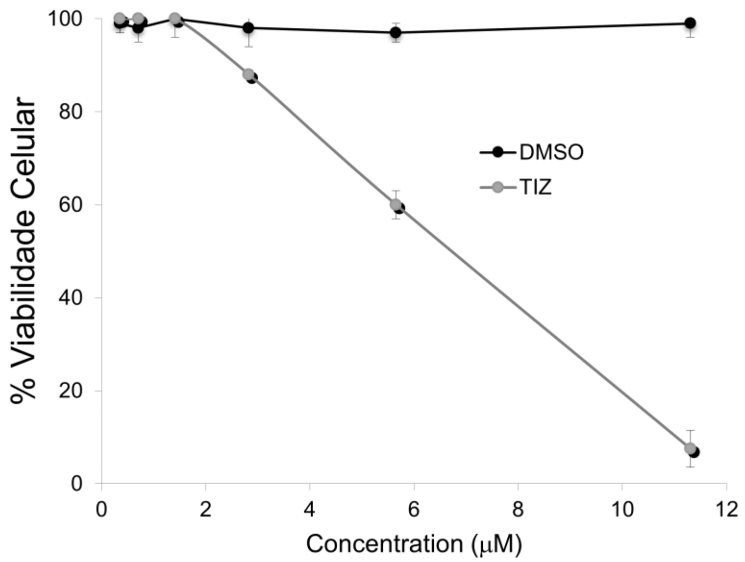

Figure S1. Viability of TIZ-treated Vero cells (72-h treatment) by neutral red dye uptake method. Data are presented as mean % cell viability of six replicates, compared to non-treated cell controls ± SD. The SD bars are obscured by the dot.
